# Peripheral Neuropathy Associated With Proteasome Inhibitors and Immunomodulatory Drugs: A Pharmacovigilance Disproportionality Analysis Using VigiBase

**DOI:** 10.1002/prp2.70191

**Published:** 2025-12-04

**Authors:** Letícia Penna Braga, Cristiane Aparecida Menezes de Pádua, Iwyson Henrique F. da Costa, Paula Lana de Miranda Drummond, Pedro Henrique Carvalho de Souza, Laura Beatriz Fonseca, Marina Alacoque Rodrigues, Jéssica Soares Malta, Adriano Max Moreira Reis

**Affiliations:** ^1^ Faculdade de Farmácia Universidade Federal de Minas Gerais Belo Horizonte Minas Gerais Brazil; ^2^ Hospital Das Clínicas da Universidade Federal de Minas Gerais Belo Horizonte Minas Gerais Brazil; ^3^ Fundação Ezequiel Dias Belo Horizonte Minas Gerais Brazil

## Abstract

Immunomodulatory drugs (IMIDs) and proteasome inhibitors (PIs) are part of the frontline treatment landscape for multiple myeloma (MM). Despite their effectiveness, these drugs are associated with adverse effects, particularly clinically significant drug‐induced peripheral neuropathy. The aim of this study was to evaluate the association between peripheral neuropathy and IMIDs and PIs used in MM, stratified by type of nerve dysfunction. VigiBase, the World Health Organization's global database of individual case safety reports (ICSRs), was analyzed. All ICSRs reported from 1 December 2001 to 31 May 2023 were extracted. Peripheral neuropathy cases were identified using MedDRA‐preferred terms (neuropathy peripheral, autonomic neuropathy, peripheral motor neuropathy, peripheral sensory neuropathy) and standardized MedDRA queries (SMQ: peripheral neuropathy). Disproportionality signals were assessed using the reporting odds ratio (ROR) and information component (IC). Associations with peripheral neuropathy were found for all IMIDs and PIs. Both IMIDs and PIs were associated with peripheral sensory neuropathy. Associations with autonomic neuropathy were observed for bortezomib (ROR 12.90; 95% CI: 9.01–18.47), ixazomib (ROR 19.01; 95% CI: 7.89–45.80), carfilzomib (ROR 9.35; 95% CI: 3.01–29.10) thalidomide (ROR 8.86; 95% CI: 4.21–18.70). Associations with peripheral motor neuropathy were detected for bortezomib (ROR 63.87; 95% CI: 51.78–78.80), thalidomide (ROR 30.62; 95% CI: 22.67–41.40), lenalidomide (ROR 2.95; 95% CI: 2.11–4.14), pomalidomide (ROR 5.03; 95% CI: 2.91–8.69). Signals of autonomic neuropathy were identified for bortezomib, carfilzomib, ixazomib, and thalidomide, while signals of peripheral motor neuropathy were observed for bortezomib, thalidomide, lenalidomide, and pomalidomide. Associations with peripheral sensory neuropathy were detected for all IMIDs and PIs analyzed.

## 1 Introduction

The frontline treatment landscape for multiple myeloma (MM) includes small‐molecule drugs and immunotherapies [1, 2, 3]. Immunomodulatory drugs (IMIDs) and proteasome inhibitors (PIs) are small‐molecule agents that inhibit myeloma cell proliferation and angiogenesis, induce apoptosis, and modulate interactions between myeloma cells and bone marrow stromal cells [2, 4]. Beyond MM, some IMIDs are approved for other conditions: thalidomide for erythema nodosum leprosum and lenalidomide for myelodysplastic syndrome and lymphomas [2].

Over the past two decades, the use of IMIDs (thalidomide, lenalidomide, and pomalidomide) and PIs‐including the first‐generation agent bortezomib and the newer agents carfilzomib and ixazomib has significantly improved survival rates in MM [3, 5]. However, despite their effectiveness, these drugs are associated with adverse effects, particularly drug‐induced peripheral neuropathy, which remains a major concern [3, 4].

Drug‐induced peripheral neuropathy is a clinically significant adverse event that compromises treatment effectiveness and patients' health‐related quality of life. In a Brazilian cross‐sectional study, 90.3% of MM patients reported chemotherapy‐induced peripheral neuropathy, with 62.7% experiencing severe symptoms and more than half reporting a substantial impact on daily activities [6]. Another study found that peripheral neuropathy led to dose reduction, discontinuation, or switching of frontline therapy in 41% and 51% of newly diagnosed MM patients, respectively. Time‐varying peripheral neuropathy was further associated with treatment delays, discontinuation, or therapy switch [7]. In addition to its clinical burden, peripheral neuropathy has been linked to increased healthcare costs in the US and Sweden, including higher hospitalization rates, greater emergency department use, and more outpatient visits [8, 9].

Peripheral neuropathy, whether drug‐induced or MM‐related, can involve sensory, motor, or autonomic nerve dysfunction [4, 5, 10]. Sensory neuropathy is the most common, manifesting as numbness, tingling, altered touch sensation, impaired vibration, paraesthesia, and dysesthesias. Motor neuropathy occurs less frequently and is characterized by distal weakness, gait and balance disturbances, and impaired movements. Autonomic neuropathy is less common and typically presents with orthostatic hypotension, reduced heart rate variability, and delayed gastric emptying [4, 5, 11].

The incidence of thalidomide‐induced peripheral neuropathy varies widely (23%–83%), depending on the study population, treatment duration, and dose. While pomalidomide and lenalidomide are associated with lower neurotoxicity, safety data from clinical trials remain limited [3, 10, 11].

A systematic review of phase III trials confirmed the substantial neurotoxicity of bortezomib [5]. Second‐generation PIs, such as carfilzomib and ixazomib, generally induce less peripheral neuropathy than bortezomib; however, real‐world safety data remain limited [5, 12, 13, 14]. Importantly, little is known about drug‐induced peripheral neuropathy stratified by type of nerve dysfunction (sensory, motor, autonomic), and the relative contribution of each IMID and PI to these subtypes remains unclear.

Spontaneous reporting systems remain pivotal for post‐marketing surveillance. VigiBase, the World Health Organization's (WHO) global database of spontaneous safety reports, provides real‐world risk assessments of drugs in large populations and supports signal detection to inform global health initiatives and risk minimization strategies. VigiBase is also widely used for disproportionality analyses, a recognized method for early signal detection increasingly employed by pharmaceutical companies, regulatory agencies, and researchers [15, 16, 17]. Because MM patients are at high risk of peripheral neuropathy, understanding this adverse event is essential to optimize prevention and management without compromising therapeutic effectiveness.

The aim of this study was to perform disproportionality analyses using VigiBase to determine the association of peripheral neuropathy with IMIDs and PIs, stratified by type of nerve dysfunction.

## 2 Methods

### 2.1 Study Design

This observational, retrospective pharmacovigilance study employed a disproportionality analysis based on the VigiBase database.

### 2.2 Data Description

VigiBase, the WHO global database of individual case safety reports (ICSRs), was used in this study. VigiBase is managed by the Uppsala Monitoring Centre (Uppsala, Sweden) and contains more than 35 million ICSRs submitted by at least 130 member countries since 1967. ICSRs originate from several sources, including healthcare professionals, patients, and pharmaceutical companies, and are typically reported in the post‐marketing phase. Each report includes administrative information (country, report type, and qualification of reporter), patient data (age and sex), date of onset of reaction(s), the nature of the outcome using Medical Dictionary for Regulatory Activities (MedDRA) version 26.0 terms, and drug(s) involved (name, start and stop dates, time to onset, indication, and dose). Drugs were coded using the WHO Drug Dictionary, which covers more than 150 000 medicines and vaccines. Each adverse drug reaction (ADR) was categorized as “serious” or “non‐serious” according to the WHO definition. The probability that a suspected adverse effect reported in VigiBase is drug‐related varies [18].

### 2.3 Data Extraction and Selection

All ICSRs from all geographic regions reported between 1 December 2001 and 31 May 2023 in VigiBase were extracted. The start date was defined as the approval of thalidomide for the treatment of MM by the European Medicines Agency on 20 November 2001 [19]. The extraction date was 21 August 2023.

All deduplicated ICSRs involving at least one PI or IMID as suspected or interacting drugs, and reported within the study period, were extracted from the Uppsala Monitoring Centre database. The Anatomical Therapeutic Chemical (ATC) classification codes for the PIs and IMIDs included in the study are shown in Box 1.

BOX 1 **Anatomical therapeutic chemical classification for drugs used in the analysis.**

**Table:** 

ATC code	Name
L01XG	Proteasome inhibitors
L01XG01	Bortezomib
L01XG02	Carfilzomib
L01XG03	Ixazomib
L04AX	Other immunosuppressants
L04AX02	Thalidomide
L04AX04	Lenalidomide
L04AX06	Pomalidomide

MedDRA‐preferred terms (PTs) describing types of nerve dysfunction related to peripheral neuropathy—including autonomic neuropathy, peripheral neuropathy, peripheral motor neuropathy, peripheral sensory neuropathy, and the standardized MedDRA query (SMQ) for peripheral neuropathy (Box 2) were used for case extraction. MedDRA version 26.0 was adopted for this study [20].

BOX 2 **MedDRA codes for preferred terms and standardized MedDRA queries used in the analysis.**

**Table:** 

MedDRA code	MedDRA‐PT/SMQ
10034580	Anatomic neuropathy
10003841	Neuropathy peripheral
10034580	Peripheral motor neuropathy
10040038	Peripheral sensory neuropathy
20000034	Peripheral neuropathy

### 2.4 Statistical Analysis

Disproportionality analysis was performed to determine whether suspected drug‐induced peripheral neuropathy was reported more frequently for PIs and IMIDs compared with peripheral neuropathy cases reported in the full database. The analysis employed both the reporting odds ratio (ROR) with 95% confidence intervals (CIs) and the information component (IC).

Because ICSR databases do not include patients who took the drug of interest without experiencing an adverse event, in the disproportionality analysis, patients experiencing the adverse event of interest were considered cases, while those experiencing all other adverse events were considered non‐cases [21]. The ROR uses a case–noncase approach to detect disproportionality signals by calculating the ratio of the odds of drug reports for a specific medication to the odds of the same adverse reaction for a control group of medications. RORs comparing drugs within each group PIs (bortezomib vs. ixazomib, bortezomib vs. carfilzomib, and ixazomib vs. carfilzomib) and IMIDs (thalidomide vs. lenalidomide, thalidomide vs. pomalidomide, and lenalidomide vs. pomalidomide) were calculated. A signal was considered disproportionate when the ROR value and the lower limit of the corresponding 95% CI were greater than 1 [21, 22, 23], with higher ROR values indicating stronger signals [22, 24].

The Bayesian IC, a statistical measure developed and validated by the Uppsala Monitoring Centre, is a flexible and automated indicator for detecting disproportionate reporting signals. Using a Bayesian confidence propagation neural network, this approach compares observed and expected drug–adverse event associations to identify new signals by quantifying probability differences from the background dataset (full database) [21, 24]. Based on information theory, probabilistic reasoning effectively manages large datasets, demonstrating robustness in handling incomplete information and complex variables. The IC is considered more accurate than the ROR when the number of cases is low [25]. The IC value represents the lower bound of the 95% credibility interval, and a positive IC value (> 0) is the standard threshold for signal detection at the Uppsala Monitoring Centre, indicating that a specific drug–adverse event combination is reported more frequently than expected [23, 24, 25]. Higher IC values indicate stronger signals [23, 26]. All analyses were conducted using SAS OnDemand for Academics.

### 2.5 Ethics Statement

The data used in this study was derived from individual case safety reports in the International Pharmacovigilance Database. Ethics approval was not required for this study.

## 3 Results

**FIGURE 1:**
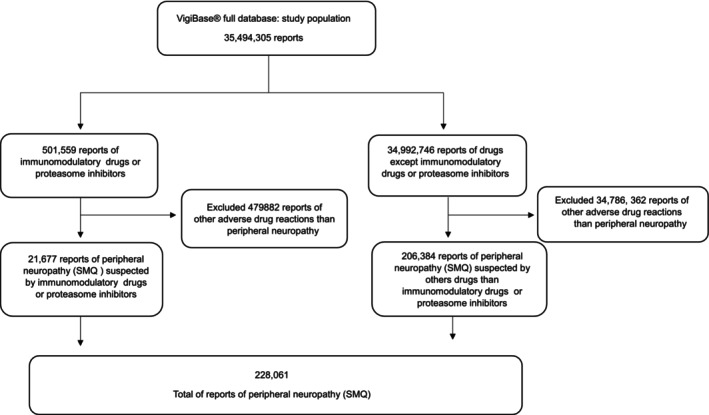
Flowchart of cases/no cases of peripheral neuropathy reported in VigiBase from December 1st, 2001 to May 31st, 2023. Date of extraction: August 21st, 2023.

Of the 35 494 304 ICSRs extracted from VigiBase, 501 559 involved at least one PI or IMID. Among these, 21 677 were reports of peripheral neuropathy based on the SMQ. Within this therapeutic group, 14 357 (66.2%) ICSRs were suspected to be associated with IMIDs and 7320 (33.8%) with PIs (Figure 1).

The number of ICSRs stratified by drug, PT, and SMQ is presented in Table 1. Among the 14 357 IMID‐related ICSRs, 9845 (68.6%) were attributed to lenalidomide. Among the 7320 PI‐related ICSRs, bortezomib accounted for 6178 (84.4%). “Peripheral neuropathy” and “peripheral sensory neuropathy” were the most frequently reported PTs across both drug classes. Autonomic neuropathy was reported less frequently, with 714 cases in the full database, of which 103 (14.4%) were suspected to be PI‐related and 20 (2.8%) IMID‐related. Bortezomib was implicated in 95 (92.2%) of the PI‐associated autonomic neuropathy cases.

**TABLE 1:** Number of reports of peripheral neuropathy associated with proteasome inhibitors and immunomodulatory drugs in VigiBase, 2001–2023.

MedDRA terms	All ICSR in VigiBase	ICSR for drugs					
		Proteasome inhibitors			Immunomodulatory drugs		
		Bortezomib	Carfilzomib	Ixazomib	Thalidomide	Lenalidomide	Pomalidomide
**Preferential term**							
Autonomic neuropathy	714	95	3	5	7	10	3
Neuropathy peripheral	102 877	4772	315	664	2049	8732	1737
Peripheral motor neuropathy	1331	94	2	1	44	35	13
Peripheral sensory neuropathy	6752	439	15	31	201	235	60
**SMQ**							
Peripheral neuropathy	228 061	6178	394	748	2561	9845	1951

Abbreviations: ICSR, individual case safety report; SMQ, standardized MedDRA queries.

**FIGURE 2:**
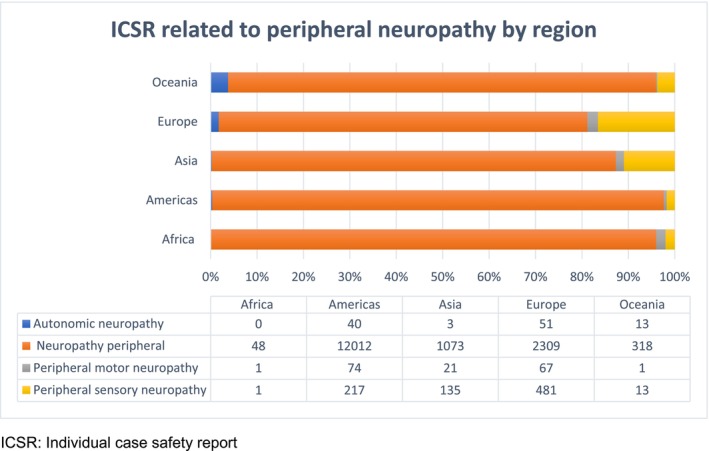
Individual case safety reports for peripheral neuropathy according to MedDRA preferred terms by global region reported in VigiBase, 2001–2023. ICSR, individual case safety report.

By geographical region, the Americas accounted for 12 356 (73.1%) of the 16 878 reported ICSRs, followed by Europe (17.2%) and Asia (7.3%) (Figure 2). Over time, the number of ICSRs increased, with peaks in 2015 (1653 reports) and 2021 (2259 reports) (Table 2). Overall, most reports were classified as serious. Serious cases of autonomic neuropathy and peripheral motor neuropathy were most frequently reported for bortezomib and thalidomide, whereas lenalidomide was most associated with serious peripheral motor neuropathy (Table 3).

**TABLE 2:** Individual case safety reports for peripheral neuropathy by year reported in VigiBase, 2001–2023.

Year	MedDRA preferred term				All terms
	Autonomic neuropathy	Neuropathy peripheral	Peripheral motor neuropathy	Peripheral sensory neuropathy	
2001	0	1	0	0	1
2002	0	50	0	1	51
2003	1	20	1	0	22
2004	0	57	2	3	62
2005	6	158	6	28	198
2006	1	188	3	11	203
2007	0	8	0	0	8
2008	0	300	8	33	341
2009	4	248	12	24	288
2010	1	290	10	30	331
2011	9	424	15	40	488
2012	10	410	11	40	471
2013	3	259	7	23	292
2014	5	1169	13	138	1325
2015	12	1573	17	51	1653
2016	7	879	6	44	936
2017	7	1108	15	54	1184
2018	9	1050	15	132	1206
2019	7	1163	4	68	1242
2020	5	1660	9	49	1723
2021	3	2218	7	31	2259
2022	5	1882	1	30	1918
2023	0	645	2	17	664
Total	107	15 760	164	847	16 878

**TABLE 3:** Seriousness of peripheral neuropathy cases reported for proteasome inhibitors and immunomodulatory drugs.

Drug	MedDRA preferred term			
Seriousness^a^	Autonomic neuropathy *n* (%)	Neuropathy peripheral *n* (%)	Peripheral motor neuropathy *n* (%)	Peripheral sensory neuropathy *n* (%)
**Proteasome inhibitors**				
*Bortezomib*				
Serious	87 (92.6)	3421 (60.7)	75 (77.3)	276 (60.8)
Non‐serious	5 (5.3)	1939 (34.4)	19 (19.6)	154 (33.9)
Unknown	2 (2.1)	278 (4.9)	3 (3.1)	24 (5.3)
Total	94 (100.0)	5638 (100.0)	97 (100.0)	454 (100.0)
*Carfilzomib*				
Serious	2 (66.7)	287 (66.1)	3 (100.0)	9 (60.0)
Non‐serious	0 (0.0)	136 (31.3)	0 (0.0)	6 (40.0)
Unknown	1 (33.3)	11 (2.5)	—	—
Total	3 (100.0)	434 (100.0)	3 (100.0)	15 (100.0)
*Ixazomib*				
Serious	3 (60.0)	561 (64.3)	1 (100.0)	12 (41.4)
Non‐serious	1 (20.0)	296 (33.9)	0 (0.0)	17 (58.6)
Unknown	1 (20.0)	16 (1.8)	—	—
Total	5 (100.0)	873 (100.0)	1 (100.0)	29 (100.0)
**Immunomodulatory drugs**				
*Thalidomide*				
Serious	8 (88.9)	1323 (66.5)	36 (78.3)	145 (69.7)
Non‐serious	0 (0.0)	496 (24.9)	8 (17.4)	47 (22.6)
Unknown	1 (11.1)	169 (8.5)	2 (4.3)	16 (7.7)
Total	9 (100.0)	1988 (100.0)	46 (100.0)	208 (100.0)
*Lenalidomide*				
Serious	7 (50.0)	7095 (80.3)	28 (80.0)	125 (47.5)
Non‐serious	2 (14.3)	1660 (18.8)	7 (20.0)	133 (50.6)
Unknown	5 (35.7)	86 (1.0)	—	5 (1.9)
Total	14 (100.0)	8841 (100.0)	35 (100.0)	263 (100.0)
*Pomalidomide*				
Serious	3 (100.0)	1397 (79.4)	9 (69.2)	33 (51.6)
Non‐serious	0 (0.0)	354 (20.1)	4 (30.8)	31 (48.4)
Unknown	—	8 (0.5)	—	—
Total	3 (100.0)	1759 (100.0)	13 (100.0)	64 (100.0)

^a^ Caused/prolonged hospitalization, disabling/incapacitating, life‐threatening, death.

Peripheral neuropathy (SMQ) signals were detected for all IMIDs and PIs compared with all other drugs. The highest ROR was observed for bortezomib (27.17, 95% CI = 26.45–27.90) and the lowest for carfilzomib (3.91, 95% CI = 3.53–4.32).

Signals for “peripheral neuropathy” and “peripheral sensory neuropathy” were observed for all MM drugs investigated. The strongest association with peripheral neuropathy was for bortezomib (ROR 45.85, 95% CI: 44.47–47.30), followed by thalidomide (ROR 19.12, 95% CI: 18.28–20.00) and ixazomib (ROR 18.39, 95% CI: 17.01–19.90). All other drugs had RORs < 18.4 (Table 4). The pattern for peripheral sensory neuropathy was similar: bortezomib showed the strongest association (ROR 58.92, 95% CI: 53.46–64.90), followed by ixazomib (ROR 12.46, 95% CI: 8.75–17.70), while all other drugs had RORs < 5.

**TABLE 4:** Disproportionality analysis for peripheral neuropathy associated with proteasome inhibitors and immunomodulatory drugs compared to all other drugs in VigiBase, 2001–2023.

Drug	MedDRA preferred term								SMQ	
	Autonomic neuropathy		Neuropathy peripheral		Peripheral motor neuropathy		Peripheral sensory neuropathy		Peripheral neuropathy	
	Reported odds ratio—ROR (95% CI)									
	ROR	95% CI	ROR	95% CI	ROR	95% CI	ROR	95% CI	ROR	95% CI
**Proteasome inhibitors**										
Bortezomib	129.00	(103.92–160.13)	45.85	(44.47–47.30)	63.87	(51.78–78.8)	58.92	(53.46–64.9)	27.17	(26.45–27.90)
Carfilzomib	9.35	(3.01–29.10)	6.92	(6.19–7.74)	3.33	(0.83–13.3)	4.93	(2.97–8.2)	3.91	(3.53–4.32)
Ixazomib	19.01	(7.89–45.8)	18.39	(17.01–19.9)	2.03	(0.28–14.4)	12.46	(8.75–17.7)	9.34	(8.68–10.06)
**Immunomodulatory drugs**										
Thalidomide	8.86	(4.21–18.70)	19.12	(18.28–20.00)	30.62	(22.67–41.4)	27.59	(23.97–31.8)	10.79	(10.37–11.20)
Lenalidomide	1.55	(0.83–2.91)	10.41	(10.19–10.70)	2.95	(2.11–4.14)	3.95	(3.47–4.50)	5.06	(10.37–11.20)
Pomalidomide	2.15	(0.69–6.69)	8.96	(8.54–9.40)	5.03	(2.91–8.69)	4.57	(3.55–5.90)	4.49	(4.30–4.71)

	Information component—IC (95% CI)									
	IC	95% CI	IC	95% CI	IC	95% CI	IC	95% CI	IC	95% CI
**Proteasome inhibitors**										
Bortezomib	6.41	(5.83–6.41)	5.27	(5.23–5.31)	5.50	(5.19–5.77)	5.68	(5.55–5.82)	4.51	(4.47–4.54)
Carfilzomib	2.09	(0.04–3.28)	2.75	(2.58–2.90)	1.18	(−1.40–2,54)	2.13	(1.31–2.76)	1.93	(1.78–2.07)
Ixazomib	2.84	(1.31–3.84)	4.10	(3.99–4.21)	0.59	(−3.20–2.23)	3.39	(2.83–3.85)	3.13	(3.03–3.24)
**Immunomodulatory drugs**										
Thalidomide	2.53	(1.27–3.41)	4.15	(4.09–4.21)	4.48	(4.03–4.88)	4.64	(4.44–4.84)	3.32	(3.27–3.38)
Lenalidomide	0.59	(−0.44–1.35)	3.23	(3.20–3.25)	1.50	(0.98–1.94)	1.93	(1.74–2.11)	2.25	(2.22–2.28)
Pomalidomide	0.88	(−1.16–2.07)	3.1	(3.03–3.17)	2.12	(1.23–2.79)	2.14	(1.75–2.48)	2.12	(2.06–2.19)

Abbreviations: 95**%** CI, 95% confidence intervals; IC, Information Component; ROR, Reported Odds Ratio; SMQ, Standardized MedDRA Queries.

Autonomic neuropathy signals were detected for PIs [bortezomib (ROR 12.90, 95% CI: 9.01–18.50), ixazomib (ROR 19.01, 95% CI: 7.89–45.8), and carfilzomib (ROR 9.35, 95% CI: 3.01–29.10)] and IMIDs [thalidomide (ROR 8.86, 95% CI: 4.21–18.70)] compared with all other drugs.

Peripheral motor neuropathy signals were observed only for bortezomib among the PIs (ROR = 63.87, 95% CI = 51.78–78.80), whereas all IMIDs were associated with peripheral motor neuropathy: thalidomide (ROR = 30.62, 95% CI = 22.67–41.4), lenalidomide (ROR = 2.95, 95% CI: 2.11–4.14), and pomalidomide (ROR = 5.03, 95% CI: 2.91–8.69).

IC analysis confirmed disproportionality for autonomic neuropathy associated with bortezomib (IC 6.41, 95% CI: 5.83–6.41), ixazomib (IC 2.84, 95% CI: 1.31–3.84), and thalidomide (IC 2.53, 95% CI: 1.27–3.41). Disproportionality was also found for peripheral motor neuropathy associated with bortezomib (IC 5.50, 95% CI: 5.19–5.77), thalidomide (IC 4.48, 95% CI: 4.03–4.88), and pomalidomide (IC 2.12, 95% CI: 1.23–2.79). Signals for peripheral neuropathy (SMQ), peripheral neuropathy, and peripheral sensory neuropathy were confirmed across all IMIDs and PIs in the IC analysis (Table 4).

The case‐noncase analysis for peripheral neuropathy (Table 5) showed that bortezomib was associated with higher reporting rates than carfilzomib and ixazomib for all types of nerve dysfunction. Ixazomib, compared with carfilzomib, showed higher reporting rates for peripheral neuropathy (ROR 2.64, 95% CI: 2.31–3.03) and peripheral sensory neuropathy (ROR 2.52, 95% CI: 1.36–4.67). Among IMIDs, thalidomide had higher reporting rates than lenalidomide or pomalidomide for all types of nerve dysfunction. Lenalidomide, compared with pomalidomide, showed a slightly higher reporting rate for peripheral neuropathy (ROR 1.09, 95% CI: 1.03–1.14).

**TABLE 5:** Reporting odds ratio (ROR) and 95% confidence interval (95% CI) comparing proteasome inhibitors and immunomodulatory drugs regarding selected peripheral neuropathy adverse events in VigiBase, 2001–2023.

MedDRA terms	Therapeutic drug class											
	Proteasome inhibitors						Immunomodulatory drugs					
	Bortezomib vs. ixazomib		Bortezomib vs. carfilzomib		Ixazomib vs. carfilzomib		Thalidomide vs. lenalidomide		Thalidomide vs. pomalidomide		Lenalidomide vs. pomalidomide	
	ROR (95% CI)	*p*	ROR (95% CI)	*p*	ROR (95% CI)	*p*	ROR (95% CI)	*p*	ROR (95% CI)	*p*	ROR (95% CI)	*p*
**Preferential term**												
Autonomic neuropathy	5.93		12.02		2.02		5.67		4.09		0.72	
	(2.41–14.57)	< 0.001	(3.81–37.94)	< 0.001	(0.48–8.49)	0.32	(2.16–14‐91)	< 0.001	(1.06–15.82)	0.003	(0.20–2.62)	0.62
Neuropathy Peripheral	2.39		6.34		2.64		1.95		2.12		1.09	
	(2.20–2.60)	< 0.001	(5.65–7.12)	< 0.001	(2.31–3.03)	< 0.001	(1.87–2.05)	< 0.001	(1.99–2.27)	< 0.001	(1.03–1.14)	0.001
Peripheral motor neuropathy	29.33		17.80		0.61		10.20		5.93		0.58	
	(4.09–210.40)	< 0.001	(4.39–72.24)	< 0.001	(0.05–6.71)	0.68	(6.54–15.90)	< 0.001	(3.19–11.02)	< 0.001	(0.31–1.10)	0.09
Peripheral sensory neuropathy	4.44		11.08		2.52		27.28		5.51		0.85	
	(3.09–6.40)	< 0.001	(6.62–18.54)	< 0.001	(1.36–4.67)	0.002	(20.44–36.40)	< 0.001	(4.13–7.35)	< 0.001	(0.64–1.12)	0.25
**SMQ**												
Peripheral neuropathy	2.84		6.78		2.39		2.18		2.39		1.09	
	(2.62–3.07)	< 0.001	(6.12–7.52)	< 0.001	(2.11–2.70)	< 0.001	(2.09–2.28)	< 0.001	(2.25–2.54)	< 0.001	(1.04–1.15)	0.003

Abbreviations: 95% CI, 95% confidence intervals; ROR, Reported Odds Ratio; SMQ, Standardized MedDRA Queries.

## 4 Discussion

This study is the first disproportionality analysis of peripheral neuropathy and drugs used in MM stratified by type of nerve dysfunction (sensory, motor, and autonomic) using the comprehensive pharmacovigilance database VigiBase. Disproportionality was identified for autonomic neuropathy induced by bortezomib, carfilzomib, ixazomib, and thalidomide using both ROR and IC. Similar findings were observed for peripheral motor neuropathy induced by bortezomib, thalidomide, lenalidomide, and pomalidomide. For neuropathy peripheral (PT), peripheral neuropathy (SMQ), and peripheral sensory neuropathy, disproportionality was detected for all IMIDs and PIs included in the study. In line with current scientific evidence, our study confirmed signals of peripheral neuropathy associated with both IMIDs and PIs, highlighting the importance of close monitoring, appropriate dose adjustments, and supportive care to mitigate neuropathic effects while allowing continued treatment [5, 11, 27, 28].

From a clinical perspective, drug‐induced peripheral neuropathy is not limited to sensory nerve damage; patients may present with symptoms or signs of motor or autonomic nervous system involvement, or a combination of both [25]. The predominant manifestation of bortezomib‐induced peripheral neuropathy is moderate to severe neuropathic pain in the distal extremities, such as the fingertips, toes, or soles [7, 24]. Autonomic neuropathy associated with orthostatic hypotension, bradycardia, and delayed gastric emptying has also been reported, which is consistent with the signal detected for bortezomib in this study [4, 29].

An association between autonomic symptoms and bortezomib use was suggested following the first reported case of bortezomib‐induced paralytic ileus [30]. Giannoccaro et al. [31] demonstrated in an immunofluorescence study that bortezomib can cause neuropathy involving small somatic and autonomic fibers in a length‐dependent manner. Another case report from 2012 described autonomic neuropathy involving both sympathetic and parasympathetic fibers [32]. An increase in the number of ICSRs for autonomic neuropathy associated with bortezomib was identified in VigiBase after 2010, and the higher ROR values in our study suggest that healthcare providers increasingly recognize and report this adverse event. Thus, adverse events related to autonomic neuropathy during bortezomib treatment should be carefully considered in real‐world practice.

Serious autonomic bortezomib‐induced peripheral neuropathy is uncommon, and life‐threatening gastrointestinal events such as paralytic ileus are rare, typically reported only in isolated case reports [30, 33] and not in clinical trials [34, 35]. However, clinical studies have described a higher incidence of bortezomib‐induced autonomic neuropathy affecting the digestive system [36], with severity closely linked to treatment response [37]. Mechanistically, bortezomib inhibits proteases in nerve terminals, leading to dysfunction of intestinal autonomic cells [36, 38], which may explain the digestive autonomic neuropathy observed. This warrants close monitoring, prompt diagnosis, and early treatment to improve patients' quality of life and prognosis [39].

Ixazomib is an oral PI that selectively binds to the 20S proteasome subunit, inhibits its chymotrypsin‐like activity, and has a shorter dissociation half‐life than bortezomib. Carfilzomib is an irreversible PI with minimal off‐target activity, which accounts for its significantly lower incidence of peripheral neuropathy compared with that of bortezomib [3, 6, 39, 40, 41, 42]. These features may also explain the lack of signal for peripheral motor neuropathy in our study. There is some evidence that neurotoxicity is related both to the efficacy of proteasome inhibition and to off‐target effects. However, more detailed clinical studies are needed to fully clarify the relative neurotoxicity profiles of PIs.

Clinical trials suggest a higher incidence of peripheral sensory neuropathy with bortezomib‐based regimens compared with ixazomib‐based regimens for the treatment of relapsed/refractory MM. Our findings regarding the ROR of peripheral neuropathy induced by ixazomib are consistent with the current scientific evidence [28, 42, 43, 44].

Constipation is an ADR related to autonomic nerve‐fiber injury that occurs rapidly and frequently in patients receiving thalidomide, as reported in several clinical trials [28, 45, 46, 47, 48, 49]. Other clinically relevant signs of thalidomide‐induced autonomic peripheral neuropathy that remain underrecognized include impairment of gastrointestinal motility and bradycardia [28, 50]. These observations help explain the signal for thalidomide‐induced autonomic peripheral neuropathy identified in VigiBase during the study period.

The ROR values for peripheral neuropathy (SMQ) induced by IMIDs align with results from phase III studies, which revealed a low neurotoxicity profile for pomalidomide‐ and lenalidomide‐induced peripheral neuropathy [10]. The signals for peripheral neuropathy induced by thalidomide are consistent with current evidence, suggesting peripheral neuropathy as a clinically important ADR associated with thalidomide [10]. A meta‐analysis reported that lenalidomide had a lower risk of severe peripheral neuropathy when combined with bortezomib compared with thalidomide plus bortezomib [5, 51]. Disproportionality analysis stratified by type of nerve dysfunction revealed a signal for lenalidomide‐induced peripheral sensory neuropathy. This finding is supported by long‐term prospective studies of lenalidomide use in which up to 50% of patients demonstrated sensory axonal neuropathy on neurophysiological evaluation [6, 52].

The association between IMIDs and peripheral neuropathy was also investigated using the FDA Adverse Event Reporting System (FAERS), with findings consistent with our results using VigiBase. Both datasets identified significant associations between IMIDs and peripheral neuropathy. Additionally, the FAERS analysis emphasized that each IMID carries a different level of peripheral neuropathy risk, highlighting the need for tailored risk management strategies based on drug type and patient demographics, particularly age and treatment indication such as MM [53].

Studies using VigiBase provide valuable insights into drug safety but are subject to certain limitations. A key limitation is reporting bias, as VigiBase relies on spontaneous reporting. Underreporting of mild or well‐known ADRs and selective overreporting of severe ADRs are common. In our dataset, serious ADRs were reported more frequently. Another limitation is that ICSRs often lack completeness, with missing data, absence of denominators, and potential confounding factors [15, 23].

Moreover, MM treatment typically involves combination regimens, complicating [51, 54] attribution of peripheral neuropathy or other ADRs to a single drug. It is often unclear whether the ADR was caused by the reported drug or by another agent in combination.

The number of ICSRs is also influenced by the structure of national regulatory systems, explaining the higher number of reports from Europe and North America. Therefore, our results may not fully represent the global burden of peripheral neuropathy. Furthermore, the studied drugs have been introduced at different times and are used in distinct clinical and regulatory contexts, which should be considered when interpreting our findings.

Despite these limitations, our study highlights the strong association of PIs and IMIDs with peripheral neuropathy in MM treatment, particularly for bortezomib and thalidomide. Through a large‐scale pharmacovigilance analysis using VigiBase, we provide the most extensive characterization to date of ADRs associated with these therapies. These findings may facilitate early risk identification, inform therapeutic decision‐making, and support regulatory pharmacovigilance efforts.

Our results underscore the importance of increased awareness and monitoring of peripheral neuropathy in clinical practice. Improved management strategies and early intervention are critical for preserving patient quality of life during MM therapy. Prospective studies are warranted to further clarify the prevalence and spectrum of peripheral neuropathy subtypes, particularly autonomic neuropathy, given its potential to cause paralytic ileus [30, 32, 55].

## 5 Conclusion

This study corroborates the association between peripheral neuropathy and both PIs and IMIDs, providing additional evidence on signals associated with different types of nerve dysfunction underlying peripheral neuropathy. Specifically, we identified a signal of autonomic neuropathy associated with bortezomib, carfilzomib, ixazomib, and thalidomide, as well as signals of peripheral motor neuropathy associated with bortezomib, thalidomide, lenalidomide, and pomalidomide. Associations with peripheral sensory neuropathy were detected for all PIs and IMIDs evaluated. These findings highlight the importance of clinician awareness and active monitoring of these ADRs in patients receiving PIs and IMIDs during treatment for MM.

## Author Contributions

L.P.B., C.A.M.P., I.H.F.C., A.M.M.R., P.L.M.D., L.B.F., M.A.R. J.S.M., P.H.C.S. contributed substantially to the conception and design of the work. And to the data collection. L.P.B., C.A.M.P., A.M.M.R., performed the analysis and interpretation of data. All the authors contributed substantially to the draft of the manuscript. The final version of the manuscript was approved by all the authors.

## Ethics Statement

The data used in this study was based on individual case safety records reported in the International Pharmacovigilance Database. Ethics approval was not obtained for this study.

## Conflicts of Interest

The authors declare no conflicts of interest.

## Data Availability

Data were extracted from VigiBase, the WHO Global Database of Individual Case Safety Reports, made available by the Uppsala Monitoring Centre (Uppsala, Sweden). Restrictions apply to the availability of the datasets generated or analyzed during the current study, used under license for the current study and are not publicly available. Requests can be made to the corresponding authors, who will submit them to UMC for permission.
